# The Effect of Deviant Workplace Behavior on Job Performance: The Mediating Role of Organizational Shame and Moderating Role of Perceived Organizational Support

**DOI:** 10.3390/bs13070561

**Published:** 2023-07-05

**Authors:** Xin Tian, Ying Guo

**Affiliations:** School of Psychology, Sichuan Normal University, Chengdu 610066, China

**Keywords:** affective events theory, deviant behavior, framework of organizational shame, organizational shame, perceived organizational support, performance

## Abstract

It is not uncommon for employees to engage in deviant workplace behavior. Several studies have been conducted on its antecedent variables and negative effects on the organization and its members. However, the effects on employees’ emotions and behavior have been ignored. According to the affective events theory and framework of organizational shame, this study examined how deviant workplace behavior affects employee performance, explored how organizational shame mediates, and investigated the role of perceived organizational support moderators. This study was completed by 435 Chinese employees in total. The results showed the following. (1) Deviant workplace behavior significantly positively predicted organizational shame and negatively predicted job performance. Organizational shame positively predicted job performance. (2) Organizational shame mediated the relationship between deviant workplace behavior and job performance, and there were gender differences in this mediating role. (3) Perceived organizational support weakened the negative effect of deviant workplace behavior on job performance. As a result, this study proves the applicability of the framework of organizational shame in a Chinese context and provides support for the affective events theory, from the perspective of actors. Furthermore, this study offers insight into how to ameliorate the negative effects of deviant workplace behavior.

## 1. Introduction

With growing uncertainty in the organizational environment, employees are experiencing a greater conflict between their work and personal lives. Combined with the conflict between informal norms and formal organizational rules, employees are highly likely to violate organizational norms and engage in deviant behavior.

Generally, deviant workplace behavior can be described as voluntary behaviors that violate important organizational norms, including behavior aimed at the organization (e.g., intentionally arriving late and leaving early, or utilizing company resources for convenience) and other colleagues (e.g., being arrogant, rude, and insulting) [1]. Deviant workplace behavior has both constructive and destructive effects. The destructive effects refer to employees repeatedly causing huge damage to both the organization and its members to achieve personal goals [2,3]. In contrast, employees can also engage in constructive deviance that is ethical and altruistic as a means of improving corporate interests or serving others [4]. As a result, deviant workplace behavior can be viewed as a “double-edged sword” and may result in ethical dilemmas.

Although previous studies have investigated deviant workplace behavior, they are insufficient to meet the needs of business management. In addition, studying the impact of deviant workplace behavior on employees’ emotions and behaviors contributes to improving the understanding of the mechanisms of deviant workplace behavior and the theoretical framework underlying them. Furthermore, it is useful for identifying the needs of employees and recognizing the potential harm caused by deviant behavior. In this way, deviant behaviors can be reduced by satisfying employees’ needs and fostering self-reflection, which is essential for corporate management.

Therefore, our study focuses on the impact of deviant workplace behavior on employees’ emotions and behavior and its mechanisms. The study’s organization is as follows: Section 1 introduces the topic and significance of this study. Section 2 presents a review of the relevant literature, theory, and hypothesis. The research methodology is outlined in Section 3. Section 4 presents the findings and results. Section 5 presents a discussion of the results. Section 6 outlines the research significance in detail. Finally, Section 7 summarizes the limitations and future directions.

## 2. Literature Review, Theory, and Hypothesis

### 2.1. Theoretical Basis

#### 2.1.1. Affective Events Theory

Deviant workplace behavior has a significant impact on emotional and behavioral responses. According to affective events theory (AET), a wide range of workplace events can elicit emotional reactions, which ultimately influence employees’ attitudes and behaviors [5]. Affective events theory does not only apply to work events implemented by organizational leaders or colleagues. As an example, mindfulness training boosts employee satisfaction and happiness [6]. Furthermore, it can be applied to the behaviors of individuals who act as implementers [7]. For instance, positive reflection interventions can reduce stress and improve health [8].

#### 2.1.2. The Framework of Organizational Shame

Organizational shame refers to feelings induced by social devaluation in response to violations of consistency norms or threats to one’s status within the organization [9]. The framework of organizational shame (FOS) proposed by Daniels and Robinson [10] offers a new perspective for understanding the elicitation and subsequent effects of shame in the workplace. This framework suggests that employees will feel shame when their behavior does not conform to their identity standards, and they attribute it to themselves. These include ethical violations (harming others emotionally or lying), social norm violations (behaving inappropriately), and poor performance [10]. Moreover, the framework outlines three motivations associated with organizational shame, including protection, repair, and defense. These motivations do not necessarily produce the same results, but rather depend on the motivation. Protective motives, which represent traditional shame processing, may result in withdrawal behaviors, such as a reduction in constructive behavior [11,12]. Under repair motivation, employees tend to compensate and make up for their mistakes, which leads to increased cooperation and performance [13]. Under repair motivation, employees tend to focus on others and become aggressive toward those who shame them. Using this framework, Xing, Sun, and Jepsen [13] reported that negative supervisory feedback was associated with employee shame, which increased employee emotional exhaustion following work and enhanced future performance.

### 2.2. Research Hypothesis

#### 2.2.1. The Effect of Deviant Workplace Behavior on Job Performance

Job performance, a key indicator in the workplace [14], is also an indicator of the impact of deviant workplace behavior on employee behavior. Studies have shown that biased organizational behavior following a transgression creates greater inequalities. This results in a decrease in decision-making quality and job performance [15]. Furthermore, according to affective events theory, it is plausible to speculate that deviant workplace behavior could affect employee behavior by triggering emotions.

#### 2.2.2. The Mediating Role of Organizational Shame

Deviant workplace behavior is a deliberate act that violates organizational norms and causes harm to the organization and its members. Organizational norms represent organizational expectations for employee identity and work standards. Employees will internalize these expectations as their conduct norms [16]. Given the above, deviant workplace behavior indicates that employees do not adhere to organizational standards. It represents deviations from organizational norms and identity standards. This leads to an increase in shame due to the exacerbation of the gap between the real self and the ideal self. The motivation of shamed employees depends on the specific situation and cultural context. Generally, people tend to redeem their image and adopt constructive behaviors in public situations [17]. The norms of the organization are open to all employees. The behavior of employees, such as late arrivals and early leaves, is often seen by members of the organization. On the other hand, unlike Western cultures, which view shame as a negative emotion related to privacy, Eastern cultures view shame as a constructive emotion with a positive meaning [18]. Thus, employees may be able to perform better at their jobs after experiencing shame in an organizational setting in an Eastern cultural context. As a potential emotion triggered by deviant workplace behavior, organizational shame may mediate the relationship between deviant workplace behavior and job performance.

Moreover, gender differences may exist in the mediating role of organizational shame. Studies have shown that women are more likely to feel shame [19]. However, the results are inconsistent [20], which may be due to the context. Social expectations are that men are more associated with agency, women are more associated with communality [21,22]. Just as Ellemers (2018) stated that “Assertiveness and performance are seen as indicators of greater agency in men, and warmth and care for others are viewed as signs of greater communality in women” (p. 277) [22]. Ellemers (2018) also stated that “In gender expectations, men’s relevant behavior is individual task performance and anticipated priorities are work, while women’s relevant behavior is care for others and anticipated priorities are family” (Table 1 on p. 281) [22]. In order to conform to gender expectations, people gradually internalize the characteristics of the gender to which they belong over time and engage in gender-consistent impression management tactics [23,24]. Such as, in the organizational context, Bolino et al. (2016) stated that “men tend to use impression management more frequently and aggressively than women” (p. 390) and “men generally use impression management to help themselves stand out from others and to acquire instrumental rewards” (p. 390) [25]. Therefore, we hypothesize that the discrepancy between men’s self-expectations and actual performance when organizational norms are violated makes them more likely to feel shame and reshapes how others see themselves through compensation (e.g., increased job performance).

#### 2.2.3. The Moderating Role of Perceived Organizational Support

It has been discussed above that deviant workplace behavior may reduce employee performance, but under which conditions can we weaken this adverse effect? In pursuit of this question, we examine the moderating role of perceived organizational support. Perceived organizational support refers to the extent to which employees perceive that the organization values and cares about their contributions and interests [26]. In accordance with reciprocity theory, the relationship between an organization and its members is influenced by reciprocity norms, since employees who perceive organizational support are likely to reciprocate appropriately [27]. Employees who receive high levels of support report high levels of job satisfaction and well-being. They are aware of their obligation to reciprocate in positive ways, such as by reducing turnover intentions and improving performance [28,29]. Employees with unfavorable perceptions of the organization believe they are not receiving adequate support and encouragement, which ultimately results in burnout and job satisfaction decline [30]. The above findings indicate that perceived organizational support may weaken the negative effects of deviant workplace behavior on job performance.

**Figure 1:**
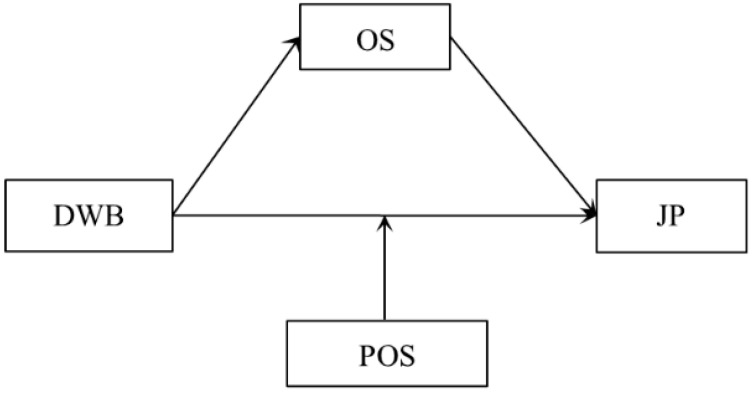
The hypothesized model. DWB = deviant workplace behavior; OS = organizational shame; JP = job performance; POS = perceived organizational support.

The above theoretical and literature review shows that preliminary studies have been conducted to investigate deviant workplace behavior, but they primarily focus on its antecedents. This includes employees’ personality traits [31,32], organizational politics [33], ethical climate [34], and leadership styles [35]. However, these findings are not sufficient to meet management practices’ requirements. A few studies on these factors have focused on their negative effects on the organization and its members, such as reducing organizational performance, organizational commitment, and colleagues’ job satisfaction [36]. However, hardly any attention has been paid to the impact on employees’ emotions and behavior. To fill this research gap, the present study combines affective events theory and the framework of organizational shame to examine the relationship between deviant workplace behavior and job performance, as well as the mediating effect of organizational shame and the moderating effect of perceived organizational support. Based on existing theoretical and previous empirical studies, we propose the following hypothesis (see Figure 1).

**Hypothesis 1**. *Deviant workplace behavior negatively correlates with job performance*.

**Hypothesis 2**. *Deviant workplace behavior positively correlates with organizational shame*.

**Hypothesis 3**. *Organizational shame positively correlates with job performance*.

**Hypothesis 4**. *Organizational shame mediates the effect of deviant workplace behavior on job performance*.

**Hypothesis 5**. *Gender differences exist in the relationship between deviant workplace behavior, organizational shame, and job performance*.

**Hypothesis 6**. *Perceived organizational support moderates the direct path of deviant workplace behavior on job performance*.

## 3. Materials and Methods

### 3.1. Participants and Procedures

A total of 458 questionnaires were distributed through WeChat groups. We collected 435 valid questionnaires from employees working in a wide range of Chinese provinces, including Sichuan, Guangxi, Chongqing, and others, with an effective rate of 94.99%. In the final sample, employees were from a variety of occupations, including sales, public relations, and human resources. Among them, 32.41% were male and 67.59% were female. The average age was 25.37 ± 1.70 years old. In addition, 82.99% had at least a bachelor’s degree.

### 3.2. Measures

#### 3.2.1. Deviant Workplace Behavior

The scale developed by Gao and Sun [37] was used. This scale has 9 items and contains both organizational transgression and interpersonal transgression dimensions on a 5-point scale (1 = very disagreeable, 5 = very agreeable). These include behaviors such as ‘leaving early and arriving late on purpose’ and ‘incivility towards colleagues.’ According to Gao and Sun, the Cronbach’s alpha coefficient for both subscales exceeded 0.87 [37]. In this study, the Cronbach’s alpha coefficient for the scale was 0.94. For organizational transgressions, it was 0.90, and for interpersonal transgressions, it was 0.94.

#### 3.2.2. Organizational Shame

The State Guilt and Shame Scale developed by Marschall et al. [38] was used. In this study, we selected the subscale of shame, which consists of five items rated on a 5-point scale from 1 = not at all to 5 = very strongly. This included statements such as ‘I want to sink into the floor and disappear.’ Existing studies have shown that the scale has good reliability and validity [39]. In this study, Cronbach’s alpha was 0.91.

#### 3.2.3. Job Performance

The scale developed by Tsui et al. [40] was used, which contains 11 questions and is scored on a 5-point scale, with 1 = totally disagree and 5 = totally agree. This included statements such as ‘the quality of work is much higher than average.’ Research has shown that the scale has satisfactory reliability and validity [41]. In this study, Cronbach’s alpha was 0.90.

#### 3.2.4. Perceived Organizational Support

The Perceived Organizational Support Scale by Shen and Benson [42] was used, which contains 8 questions on a 5-point scale, with 1 = strongly disagree to 5 = strongly agree. This included statements such as ‘my organization cares about my opinions.’ Several studies have confirmed the satisfactory reliability and validity of the scale [43]. In this study, Cronbach’s alpha coefficient was 0.73.

### 3.3. Statistical Analysis

SPSS 24.0 was used for descriptive statistics and correlation analysis, and AMOS 22.0 was used to examine the moderated mediation effect.

## 4. Results

### 4.1. Common Method Bias

The Harman’s single-factor test showed that the unrotated exploratory factor analysis had extracted six factors with characteristic roots greater than one, with a variance explained by the maximum factor variance of 7.12%, which is below the 40% threshold [44].

In addition, confirmatory factor analysis was used to test discrimination validity. We compared the single-factor model and the four-factor model (deviant workplace behavior, organizational shame, perceived organizational support, and job performance). It was found that the four-factor model (χ^2^/df = 2.72, CFI = 0.89, TLI = 0.88, RMSEA = 0.06, SRMR = 0.06) was significantly better than the single-factor model (χ^2^/df = 10.51, CFI = 0.34, TLI = 0.36, RMSEA = 0.15, SRMR = 0.20). Therefore, there was no significant common method bias.

### 4.2. Descriptive Statistics and Correlation Analysis

The results showed (Table 1) that deviant workplace behavior was significantly positively associated with organizational shame (*r* = 0.32, *p* < 0.01) and negatively associated with job performance (*r* = −0.10, *p* < 0.01). Organizational shame was significantly positively associated with job performance (*r* = 0.10, *p* < 0.01).

### 4.3. The Mediation Effect Test

According to Weijters and Baumgartner [45] and Chongming et al. [46], using the original items from the scale directly causes large parameter estimation bias. Therefore, the current study packaged organizational shame and job performance into three indicators. To reduce multicollinearity, the independent and moderating variables were centralized [47]. After controlling for age, education, occupation, and working years, the direct predictive effect of deviant workplace behavior on job performance was examined. The results showed a good model fit with χ^2^/df = 1.23, RMSEA = 0.02, CFI = 0.99, TLI = 0.99, SRMR = 0.03. Deviant workplace behavior had a significant negative predictive effect on job performance (β = −2.15, *p* < 0.05). After adding organizational shame as a mediating variable, the model fitted well with χ^2^/df = 1.30, RMSEA = 0.03, CFI = 0.99, TLI = 0.99, SRMR = 0.03. Deviant workplace behavior positively predicted organizational shame (β = 0.31, *p* < 0.001), and negatively predicted job performance (β = −0.18, *p* < 0.01). Organizational shame positively predicted job performance (β = 0.17, *p* < 0.01). According to bootstrap results, the 95% CI of deviant workplace behavior on organizational shame ([0.18, 0.43]) and job performance ([−0.28, −0.06]) did not contain zero, and the 95% CI of organizational shame on job performance ([0.05, 0.26]) did not contain zero. The results indicate that organizational shame is a mediator between deviant workplace behavior and job performance, with a mediating effect size of 0.10 and a mediating effect percentage of 30.37%.

Next, we tested the cross-gender consistency of organizational shame’s mediating effects. Two models of the mediating effects of deviant workplace behavior on job performance were tested separately for men and women. For both, the model fitted well (men: χ^2^/df = 0.06, RMSEA = 0.00, CFI = 1.00, TLI = 1.03, SRMR = 0.02; women: χ^2^/df = 1.93, RMSEA = 0.06, CFI = 0.99, TLI = 0.96, SRMR = 0.03) and it was suitable for multiple group comparisons. Therefore, gender differences were included in the mediation model and multiple comparisons were conducted to test for gender differences. The results showed that the chi-square difference between Model 1 (free estimation model) and Model 2 (equal restriction path coefficients) was significant (Δχ^2^ = 18.45, *p* < 0.05), and the fit indices ΔCFI and ΔTLI were both greater than 0.01, indicating gender differences in this mediation model.

**Figure 2:**
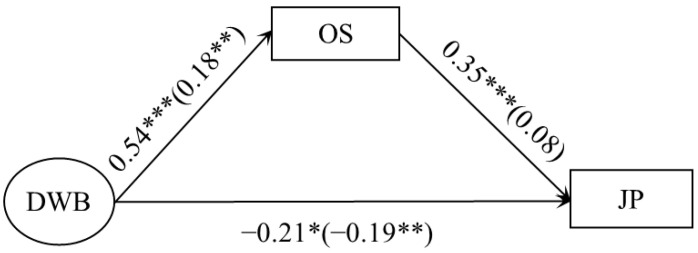
Gender differences in the mediation model. The outside brackets are male, and the inside brackets are female. Notes. * *p* < 0.05, ** *p* < 0.01, *** *p* < 0.001.

In comparison between the male and female models (see Figure 2), only the male model showed a significant mediating effect. Specifically, organizational shame significantly predicted job performance in men, but not in women. Through the bootstrap method, 2000 samples were sampled from each model to estimate the mediation effect. It was found that the mediation effect value of organizational shame was 0.19 in the male group with a 95% CI [0.10, 0.36], but it was not significant in the female group.

### 4.4. The Moderated Mediation Effect Test

**Figure 3:**
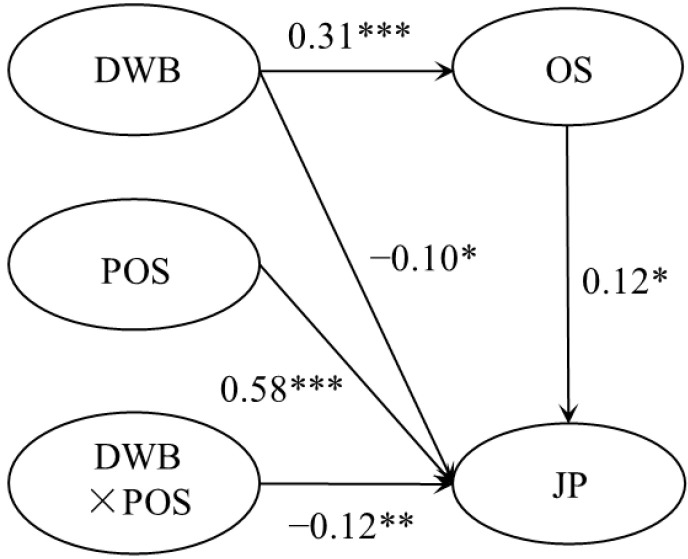
The result of the moderated mediation effect. Notes. * *p* < 0.05, ** *p* < 0.01, *** *p* < 0.001.

With the addition of organizational shame, perceived organizational support, and the interaction term between perceived organizational support and deviant workplace behavior (Figure 3), the model fitted well with χ^2^/df = 1.92, RMSEA = 0.05, CFI = 0.98, TLI = 0.96, SRMR = 0.05. The interaction term had a significant predictive effect on job performance (β = −0.12, *p* < 0.01). Bootstrap test results showed that the 95% CI of the interaction term on job performance was [−0.24, −0.02]. It indicated that the moderating effect of perceived organizational support is significant. In addition, perceived organizational support weakened the negative effect of deviant workplace behavior on job performance.

**Figure 4:**
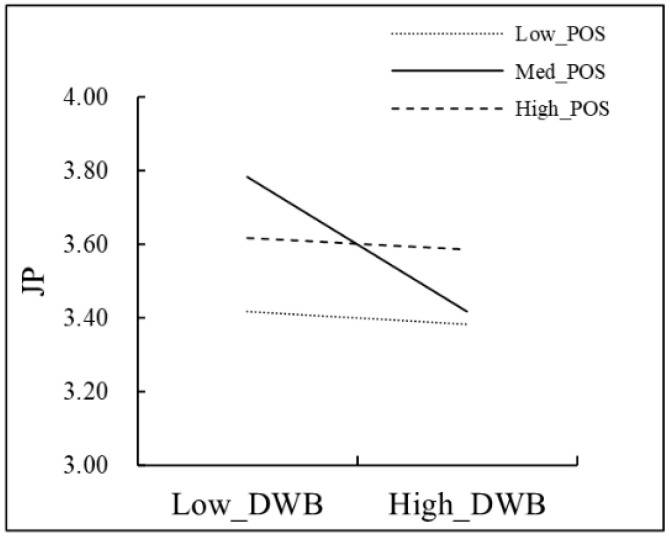
Perceived organizational support moderated the effect of deviant workplace behavior on job performance.

A further simple slope analysis (Figure 4) was conducted, with one standard deviation above being the high perceived organizational support group, equal to one standard deviation being the medium perceived organizational support group, and one standard deviation below being the low perceived organizational support group. According to the results, deviant workplace behavior had no significant predictive effect on job performance when perceived organizational support was high (β = −0.04, *t* = 0.50, *p* = 0.48) and low (β = −0.02, *t* = −0.12, *p* = 0.91). In contrast, it had a significantly negative effect on job performance when perceived organizational support was average (β = −0.19, *t* = −2.84, *p* < 0.01). This result validated the direct path of perceived organizational support in moderating the effect of deviant workplace behavior on job performance.

## 5. Discussion

Deviant workplace behavior is a common phenomenon in the workplace and has a significant impact on behavioral responses and psychological feelings of both the organization and its members. The impact on employees’ emotions and behaviors has, however, not been adequately considered in previous studies [48]. In this study, we examined the effects of deviant workplace behavior on organizational shame and job performance and explored the mediating role of organizational shame and the moderating role of perceived organizational support. Using the current study, we provide empirical evidence for both the affective events theory and the framework of organizational shame. We also provide some practical suggestions to reduce the negative effects of workplace deviance.

### 5.1. The Mediating Role of Organizational Shame and Gender Differences

This study found that deviant workplace behavior is positively related to organizational shame and negatively related to job performance, while organizational shame is positively related to job performance. Organizational shame played a mediating role between deviant workplace behavior and job performance. This provided empirical support for the affective events theory and framework of organizational shame.

According to the affective events theory, emotions play a significant role in the relationship between deviant workplace behavior and its consequences [5]. In the workplace, uncivil behavior can generate emotional responses, such as anger and guilt, that can affect employees’ psychological and behavioral responses [49]. In the organizational shame framework, it is proposed that organizational shame plays an important role in the relationship between violations and job performance. From the perspective of moral psychology, individuals usually conform to social norms to gain group approval and belonging [16]. The violation of these norms triggers shame, which is a sign that individuals are socially adaptive. By doing this, the offender is able to reintegrate back into the group, which promotes group cohesion, and increases the likelihood of reciprocal relationships [50]. Based on the results of this study, the above perspective applies equally to organizational settings. Employees who are expected to conform to the norms are more likely to feel shame when their behavior violates the minimum normative standards (e.g., harassment, cheating, etc.). As a result of organizational shame, they feel that their social self has been compromised, which motivates them to compensate for it by self-regulating and performing at a high level at work [9]. Additionally, the employees’ selves and the organization’s selves are interdependent and partially overlapped in Eastern cultures, and they emphasize harmonious relationships between themselves and others. They are more sensitive to organizational shame, and therefore they tend to demonstrate compensatory behaviors to comply with the organization’s expectations and norms [9,51].

There was also a significant gender difference in the mediating role of organizational shame. Specifically, males were more likely to improve their job performance due to organizational shame. It may be due to that society expects men to be more agency, such as achievement-oriented (achievement-focused, ambitious, etc.), and encourages them to succeed in the economy [21,22]. After internalizing the above gender characteristics, men are more concerned with their image in the workplace than women and are more likely to use impression management tactics consistent with gender role expectations [25]. In order to avoid discrepancies between social expectations, self-expectations, and actual performance, men tend to compensate and improve their performance after feeling shame for violating organizational norms. In addition, most of the subjects of our study are newcomers. In the early stages of their careers, men and women earn similar salaries. As people gain more experience and work years, their earnings increase gradually, and the gender pay gap becomes more significant [52]. For example, regarding sources of performance, Rotman and Mandel (2023) stated that “men benefited from their experience substantially more than women, and the findings are similar whether it is relative or absolute returns being considered” (p.596) [53]. Furthermore, Ellemers [22] stated that ‘women are less likely than men to be selected for promotions and prestigious positions’ (page 279). Thus, for men, their performance in the first few years determines the likelihood of future promotions and salary increases to a greater extent. Therefore, they are more likely to improve their performance after shame.

### 5.2. The Moderating Role of Perceived Organizational Support

We found that perceived organizational support can weaken the direct effect of deviant workplace behavior on job performance. According to reciprocity norms, employees who perceive organizational support are obliged to help the organization. This results in greater organizational involvement and participation in organizational activities, ultimately improving in-role and out-of-role performance [54,55]. In this regard, employees who perceive organizational support are more likely to perform well after deviant workplace behavior. Organizational support theory (OST) also suggests that organizational support helps satisfy employees’ socio-emotional needs. Employees who perceive organizational support attribute the favorable benefits they receive to attention from the organization. In order to attain a good balance in their relationship with the organization, they will develop attitudes and behaviors that align with the organization [55].

## 6. Theoretical and Practical Significance

### 6.1. Theoretical Significance

Firstly, this study supports and expands the affective events theory. According to this study, deviant workplace behavior can evoke organizational shame, which enhances employee performance. It differs from existing studies that examine the antecedent variables or negative effects of deviant workplace behavior on colleagues or the organization. This study examines the emotional and behavioral effects and consequences of deviant workplace behavior on employees. In addition to strengthening and extending the affective events theory, this study provides a comprehensive and in-depth understanding of the effects of deviant workplace behavior.

Secondly, this study contributes to an understanding of the antecedents and consequences of organizational shame in Eastern cultures. It is important to note that more researchers are drawing attention to shame in the workplace [16]. However, previous studies focused mostly on Western cultures. By examining shame in organizational settings, the present study validates the applicability of the framework of organizational shame in Eastern cultural contexts for the first time. Organizational shame can be viewed as a new perspective on how deviant workplace behavior impacts job performance, which enhances our understanding of the antecedents and consequences of organizational shame in Eastern cultures. The inclusion of organizational shame in the analysis extends the research perspective for future investigations.

Finally, the current study enhances and deepens our understanding of the boundary role of deviant workplace behavior on job performance. Employees who perceive organizational support are likely to behave in a compensatory manner after being shamed for their negligent behavior to compensate for their negative consequences. We believe that this result enriches and deepens our understanding of the boundary role. It also supports the view that perceived organizational support has a constructive function in improving the negative effects of deviant workplace behavior.

### 6.2. Practical Significance

Firstly, managers can modify or strengthen the employee code of conduct norms or the company’s mission and core values, as well as integrating online and offline training and assessment methods to facilitate employee understanding of the corporate philosophy. To avoid feeling shamed, employees must adhere to the norms consistently, thus strengthening their consistent tendency to do so.

Secondly, shame broadcasts strong signals about social norms, and people can learn social norms from it [56]. When people know that others are ashamed of certain behaviors, they assume that these behaviors are not in accordance with social norms. To avoid shame, individuals will become more compliant with social norms and adjust their behavior accordingly to conform to social norms [56,57]. Accordingly, we suggest that managers can attempt to present shame expressions that may result from violating organization norms in induction training, which include shame emotions and shame statements. Shame expressions can be expressed through emojis, internet images, or other forms, but they should not come from company employees. In this case, the company can convey its disapproval of the behavior. Considering the potential negative consequences of shame, we do not recommend managers publicly shame employees in a way similar to exposing their violations, which is disrespectful and unethical [58]. In addition, organizations can provide training on emotional management to help employees cope with shame after a violation in an adaptive manner.

Finally, the moderating effect of perceived organizational support suggests managers can create a supportive work environment by acknowledging employees’ contributions, implementing employee assistance programs, increasing procedural fairness, and using other methods to meet employees’ needs for material and spiritual aspects, such as pay, benefits, recognition, and appreciation, and thereby reduce the negative impact of transgressions on job performance.

## 7. Limitations and Future Directions

Unlike previous studies, this study examines the effects of deviant workplace behavior on employee emotions and behavior, which is novel. However, the following shortcomings still need to be addressed. Firstly, this study used a questionnaire method for cross-sectional research, so there is the possibility of reverse causality between variables, which is one aspect of this paper that is difficult to interpret. In the future, researchers may use experimental methods or longitudinal research designs to clarify causal relationships between variables. Secondly, the respondents in this study are mostly newcomers. It needs to be further explored whether these findings can be applied to a wider range of employees. Thirdly, the study only examined deviant workplace behavior’s effects on employee emotions and behavior. Future research could examine deviant workplace behavior’s effects on individual cognition, beliefs, or attitudes toward work. Finally, it is worth noting that, in addition to the variables involved in this study, studies investigating variables such as psychological well-being, organizational commitment, and work engagement may provide empirical support for affective events theory and the framework of organizational shame. Future research may further explore this in depth.

## Figures and Tables

**Table 1:** Descriptive statistics and correlation analysis.

	M	SD	1	2	3	4
1. Deviant Workplace Behavior	2.24	0.95	1			
2. Organizational Shame	2.87	1.04	0.32 **	1		
3. Job Performance	3.64	0.63	−0.10 *	0.10 *	1	
4. Perceived Organizational Support	3.02	0.58	−0.07	0.09	0.46 **	1

Notes. * *p* < 0.05, ** *p* < 0.01.

## Data Availability

The data presented in this study are available on request from the corresponding author.
